# Intimate partner violence, suicidality, and self-harm: a probability sample survey of the general population in England

**DOI:** 10.1016/S2215-0366(22)00151-1

**Published:** 2022-07

**Authors:** Sally McManus, Sylvia Walby, Estela Capelas Barbosa, Louis Appleby, Traolach Brugha, Paul E Bebbington, Elizabeth A Cook, Duleeka Knipe

**Affiliations:** a Violence and Society Centre, City, University of London, London, UK; b National Centre for Social Research, London, UK; c Division of Psychology and Mental Health, University of Manchester, Manchester, UK; d Department of Health Sciences, University of Leicester, Leicester, UK; e Division of Psychiatry, University College London, London, UK; f Population Health Sciences, Bristol Medical School, University of Bristol, Bristol, UK

## Abstract

**Background:**

Intimate partner violence (IPV) is a recognised risk factor for psychiatric disorders. There is little current evidence on IPV and self-harm and suicidality, and we therefore aimed to investigate the associations between experience of lifetime and past-year IPV with suicidal thoughts, suicide attempt, and self-harm in the past year.

**Methods:**

We analysed the 2014 Adult Psychiatric Morbidity Survey, a cross-sectional survey of 7058 adults (aged ≥16 years) in England, which used a multistage random probability sampling design and involved face-to-face interviews. Participants were asked about experience of physical violence and sexual, economic, and emotional abuse from a current or former partner, and about suicidal thoughts, suicide attempts, and self-harm. Other adversities were recorded through an adapted version of the List of Threatening Experiences. Multivariable logistic regression models quantified associations between different indicators of lifetime and past-year IPV, with past-year non-suicidal self-harm, suicidal thoughts, and suicide attempts. All analyses were weighted.

**Findings:**

Using weighted percentages, we found that a fifth (21·4%) of 7058 adults reported lifetime experience of IPV, and that 27·2% of women and 15·3% of men had experienced IPV. Among women, 19·6% had ever experienced emotional IPV, 18·7% physical IPV, 8·5% economic IPV, and 3·7% sexual IPV, which was higher than in men (8·6%, 9·3%, 3·6%, and 0·3%, respectively). Findings for ethnicity were unclear. Lifetime prevalence of IPV was higher in those living in rented accommodation or deprived neighbourhoods. Among people who had attempted suicide in the past year, 49·7% had ever experienced IPV and 23·1% had experienced IPV in the past year (including 34·8% of women and 9·4% of men). After adjusting for demographics, socioeconomics, and lifetime experience of adversities, the odds ratio of a past-year suicide attempt were 2·82 (95% CI 1·54–5·17) times higher in those who have ever experienced IPV, compared with those who had not. Fully adjusted odds ratios for past-year self-harm (2·20, 95% CI 1·37–3·53) and suicidal thoughts (1·85, 1·39–2·46) were also raised in those who had ever experienced IPV.

**Interpretation:**

IPV is common in England, especially among women, and is strongly associated with self-harm and suicidality. People presenting to services in suicidal distress or after self-harm should be asked about IPV. Interventions designed to reduce the prevalence and duration of IPV might protect and improve the lives of people at risk of self-harm and suicide.

**Funding:**

UK Prevention Research Partnership.

## Introduction

Intimate partner violence (IPV) is defined by WHO as physical violence, sexual, emotional or psychological abuse, and controlling behaviours within an intimate relationship.^1^ IPV is more prevalent in women than in men and is a known risk factor for subsequent psychiatric disorders.^2^

Although some studies have considered the relationship between specific types of IPV and suicidality or self-harm,^3^, ^4^ few have examined the wider range of IPV types with these outcomes.^5^ Existing studies are not generalisable to national, general populations as they use non-random samples and focus on subgroups:^6^ patients,^7^ service users,^8^ or young or narrow age-groups.^9^, ^10^ Most of this research focused on women only, preventing comparison with men. The WHO multi-country study using population-based surveys showed that women with experience of physical or sexual violence were nearly 4 times more likely to attempt suicide than women without such experiences, but it provided no associations for men.^11^ A 2013 systematic review found two studies of men showing an association between IPV and depressive symptoms, but no evidence for an association between IPV and subsequent suicide attempt.^10^ Methodological flaws limited these studies with men.^10^

Since the mid-1990s, three-quarters of suicides in England and Wales each year have been in men.^12^ With male rates higher in most countries,^13^ national suicide prevention strategies tend to focus on men at risk.^8^ For particular risk factors to be prioritised in such strategies, there needs to be evidence of an association with suicidality, the risk needs to be modifiable and responsive to scalable intervention, and data need to be available for monitoring progress.^14^ In calculating the costs of domestic violence, Walby drew on multiple sources to estimate that IPV could be key (alongside the cumulative effect of other adversities) in an eighth of suicides and suicide attempts.^15^

**Research in context**

**Evidence before this study**

Intimate partner violence (IPV) is known to affect the mental health of victims. However, systematic reviews done in 2012 and 2013, and a search of PubMed and Google Scholar done to update these on March 1, 2022 (using the search terms “intimate partner violence”, “suicidality,” and related terms in English; a full list of the search terms is available in the appendix [p 2]), revealed little on the impact of IPV on self-harm and suicidality. Existing studies are not generalisable to national populations (being restricted to women, young people, or other subgroups), do not focus on all aspects of IPV (often leaving out emotional and economic abuse), and rarely adjust for the wider adversities often faced by those with experience of IPV.

**Added value of this study**

Using the 2014 Adult Psychiatric Morbidity Survey of people in England, we found that approximately half of people aged 16 years and older who attempted suicide in the past year had experienced IPV at some point in their lifetime, and about one in four had experienced IPV in the preceding year (compared with one in 25 people in the rest of the population). Women were far more likely than men to experience IPV. Among women who attempted suicide, one in three had experienced IPV in the past year, and among men who attempted suicide, it was one in ten. Even with adjustment for a wide range of other adversities and demographic and socioeconomic factors, both in men and in women, the odds of suicidal thoughts, suicide attempts, and non-suicidal self-harm were all higher in those who had ever experienced IPV than in those who had not. Sexual IPV was 10 times more common in women than men, and this IPV type was associated with particularly high odds of self-harm and suicidality. Our analysis of robust health survey data indicates that IPV could be more common in England than official estimates, which are based on crime surveys.

**Implications of all the available evidence**

There is a high likelihood that someone presenting to services in suicidal distress is a victim of IPV. Health, social care, and welfare professionals should ask people who have self-harmed or are at risk of suicide if they are experiencing IPV, and professionals should be prepared, and supported, to act accordingly. Strategies for violence reduction should form part of individual-level suicide risk assessment and safety planning, and they should feature in national suicide prevention strategies.

This paper focuses on suicidal thoughts, attempts, and non-suicidal self-harm in the past year, which are some of the strongest predictors of subsequent suicide and therefore key to prevention.^14^ It also provides urgently needed data on IPV prevalence in England including in those aged 75 years and older, a group not covered in the official domestic violence prevalence estimates, which are derived from the Crime Survey for England and Wales (CSEW).

Our aims were: to estimate the prevalence of being subject to different types of violence and abuse from an intimate partner (physical, sexual, economic, or emotional) in the past year and across the life course, in women, men, and the whole adult population; to compare the prevalence of past-year non-suicidal self-harm, suicidal thoughts, and suicide attempts in people with and without experience of different types of IPV and any IPV; and to examine whether any such associations between IPV and past-year self-harm, suicidal thoughts, and suicide attempts persist after adjustment for a wide range of potential confounders, including socioeconomic context and experience of multiple other life adversities.

## Methods

### Study design and participants

We analysed the 2014 Adult Psychiatric Morbidity Survey (APMS), a cross-sectional probability-sample survey that covered the household population of England aged 16 years and older, using a stratified, multistage random sampling design, based on the Small User Postcode Address File. This involved the selection of primary sampling units, addresses within selected primary sampling units, and one adult from each address. Participants had to be able to speak English sufficiently well to be interviewed in English. Fieldwork occurred from May, 2014, to September, 2015, with verbal informed consent. The achieved sample comprised of 7546 individuals, reflecting a 57% response rate.

Interviews were done in people's homes (or elsewhere, if preferred) by trained research interviewers, and averaged 1·5 h. They involved computer-assisted personal interviewing, with some sensitive information collected through computer-assisted self-completion interview in which participants used the interviewer's laptop. The original survey was approved by the West London National Research Ethics Committee (14/LO/0411, RIT0985, 139324). The secondary analyses were approved by the committee at City, University of London that considers medium risk applications (ETH21220–299). The authors assert that all procedures contributing to this work comply with the ethical standards of the relevant national and institutional committees on human experimentation and with the Helsinki Declaration of 1975, as revised in 2008. Further methodological details are published elsewhere.^16^

### Outcomes

The primary outcomes were the prevalence of past-year non-suicidal self-harm, suicidal thoughts, and suicide attempts.

### Measures

To measure IPV, experience of physical violence and sexual, economic, and emotional abuse from a current or former partner was asked about in the self-completion section of the interview. The questions were adapted from the Crime Survey for England and Wales, originally based on the widely used Conflict Tactics Scale.^17^ Participants who had never had a partner were coded as having not experienced IPV.

Physical IPV was indicated if either of the following were endorsed: “pushed you, held or pinned you down, or slapped you” or “kicked you, bit you, or hit you with a fist or something else, or threw something at you that hurt you”. Resulting injury was established through asking: “have you ever been injured (even if only slightly) as a result of the force used on you? By injured we mean things such as bruises, black eyes, cuts or scratches, or broken bones.” Variables were derived for having experienced any physical violence from a partner, and physical violence from a partner that resulted in physical injuries. Sexual IPV was recorded if the participant responded yes to: “since the age of 16, has anyone touched you, or got you to touch them, in a sexual way without your consent?” or “since the age of 16, has anyone had sexual intercourse with you without your consent?”, or both, and if the perpetrator was a current or former partner. Variables were derived for having experienced any sexual violence from a partner (combining rape and non-consensual sexual contact) and specifically for rape. Economic IPV was recorded if the participant reported that a partner had “prevented you from having your fair share of the household money”. Emotional IPV was identified where either of the following were endorsed: “repeatedly belittled you to the extent that you felt worthless” or “sent you more than one unwanted letter, email, text message, or card that was either obscene or threatening and which caused you fear, alarm, or distress”. Variables were created for lifetime experience of each IPV type, the number of types of IPV ever experienced, experience of any IPV ever, and experience of any IPV in the past year.

For measuring suicidal thoughts in the face-to-face section of the interview, participants were asked: “have you ever thought of taking your life, even though you would not actually do it?” and when this last occurred. A variable was derived indicating such thoughts in the past year. Intentionality is complex, and suicide attempts and non-suicidal self-harm were examined separately, based on participants' own designation at the time of interview. Suicide attempts were asked about in both the face-to-face and self-completion sections with the same question: “have you ever made an attempt to take your life, by taking an overdose of tablets or in some other way?” A variable was derived that combined reports of a suicide attempt in the past year in either section. Non-suicidal self-harm, referred to here as self-harm and in some countries as self-injury, was also asked both face-to-face and in the self-completion: “have you ever deliberately harmed yourself in any way but not with the intention of killing yourself?” Self-harm in the past year drew on both the face-to-face and self-completion sections.

To take into account other adversities, an adapted version of the List of Threatening Experiences was used,^18^ which presents a range of experiences known to predict poor mental health. Participants were handed a showcard and asked for the number of the items they had experienced. Adversities experienced were summed to produce a count representing the extent to which participants had faced multiple different difficulties in their lives. 13 types were counted: serious illness or injury, serious illness or injury to a close relative, serious assault of a close relative, death of an immediate family member, death of a close family friend or other relative, violence at work, homelessness, redundancy or being sacked from a job, extended work search without success, major financial crisis, something valued being lost or stolen, having trouble with the police involving court appearance, and serving time in prison. The count was banded accordingly (0, 1–2, 3–4, or ≥5 adversities).

In order to ascertain demographic, socioeconomic and area-level factors, participants self-reported gender (women or men), age (banded for analysis), and marital or cohabitation status (single; married or cohabiting; or separated, divorced, or widowed). Ethnicity was self-ascribed and grouped into White British; White other; Black or Black British; Asian or Asian British; and mixed, multiple, or other. Socioeconomic context included housing tenure (owner-occupier, renting from social landlord, or renting from private landlord) and employment status (employed, unemployed, or economically inactive). Area-level deprivation was measured using quintiled English Index of Multiple Deprivation scores.

### Statistical analysis

Analyses were weighted and accounted for complex design, selection probabilities (likelihood that someone would be selected to take part), and non-response (refusals and non-contacts from those selected; appendix p 3). Population control totals drew on the UK Office for National Statistics population estimates for age by sex and region. Unweighted bases are presented. The prevalence of self-harm, suicidal thoughts, suicide attempts, and social circumstances were produced for those with and without lifetime experience of IPV. The statistical significance of differences between groups was indicated with a p value generated through unadjusted binary logistic regressions and by reviewing whether 95% CIs overlapped.

We did descriptive analyses on the whole sample as well as gender-stratified (given the epidemiology of suicidality^19^ and IPV differs between men and women^20^). We examined the extent to which associations between each IPV indicator and past-year self-harm and suicidality could be explained by other factors. A series of logistic regression models were run to produce unadjusted and adjusted odds ratios (OR) for each dependent variable (past-year non-suicidal self-harm, suicidal thoughts, and suicide attempt). Model version A of the multivariable regression model included experience of IPV (either any lifetime IPV, each type of lifetime IPV, number of types of lifetime IPV, or past-year IPV) as an independent variable with adjustment only for characteristics not on the causal pathway: gender, age, and ethnicity. Model version B adjusted for those demographic factors alongside further adjustment for social and socioeconomic characteristics: marital status, housing tenure, and area-level deprivation. Finally, model version C included the adjustments in model versions A and B as well as adjusting for the number of other life adversities experienced, placing IPV into a context of diverse and potentially interrelated experiences. Adjustment variable selection was informed by the rationale set out by Bandara and colleagues.^20^ Marital status, tenure, area-level deprivation, and adversities could plausibly be confounders, contributing to both IPV and suicidality. Another hypothesis is that they mediate an association between IPV and subsequent suicidality: IPV could contribute to relationship breakdown and homelessness, which in turn could lead to suicidality. If the latter, model versions B and C might over-adjust. Adjustment for victims' use of alcohol was avoided, given the implied victim-blaming. Given that suicidality is a diagnostic criterium for some mental disorders, mental health was also not adjusted for.

Interaction terms were tested to check whether the patterns of association between each IPV indicator and each suicidality and self-harm outcome differed between men and women, to inform whether gender-stratified modelling was appropriate. Participants with missing data (mostly due to participants not doing the self-completion part) were excluded from analysis. Older participants were more likely than younger ones to not do the self-completion part, which is examined elsewhere.^21^ Analyses were done in SPSS (version 21.0) and Stata (version 14.1).

### Role of the funding source

The funder of the study had no role in study design, data collection, data analysis, data interpretation, or writing of the report.

## Results

The APMS sample included 7546 individuals. Missing data mostly resulted from participants not doing the self-completion part of the survey: 462 participants did not respond to the questions on experience of IPV because of this. A further 20 did the self-completion part but did not respond to these items, and 6 responded don't know. They were excluded from analyses, yielding an analytic sample of 7058.

In 2014, approximately one in five adults in England had ever experienced violence or abuse from an intimate partner at some point in their life (1702 [21·4%] of 7058; table 1). It should be noted that all percentages are weighted rather than derived from the raw base sizes, as described earlier. Lifetime experience of any IPV was more common in women (1243 [27·2%] of 4183) than in men (459 [15·3%] of 2875).

**Table 1:** Characteristics of people with and without lifetime experience of IPV

			**No IPV**	**IPV ever**	Total*	**p value**†
Total*			5356 (78·6%)	1702 (21·4%)	7058 (100%)	..
Gender			..	..	..	<0·001
	Men		2416 (52·8%)	459 (35·2%)	2875 (49·1%)	
	Women		2940 (47·2%)	1243 (64·8%)	4183 (50·9%)	..
Age, years			..	..	..	<0·001
	16–34		1130 (31·5%)	416 (32·9%)	1546 (31·8%)	
	35–54		1650 (32·0%)	714 (40·7%)	2364 (33·9%)	..
	55–74		1777 (26·2%)	491 (22·8%)	2268 (25·5%)	..
	≥75		799 (10·2%)	81 (3·6%)	880 (8·8%)	..
Ethnic group			..	..	..	0·007
	White British		4529 (80·2%)	1481 (84·7%)	6010 (81·2%)	
	White other		321 (7·0%)	76 (5·4%)	397 (6·6%)	..
	Black or Black British		132 (3·1%)	47 (3·0%)	179 (3·0%)	..
	Asian or Asian British		266 (7·5%)	55 (4·4%)	321 (6·8%)	..
	Mixed, multiple, or other		97 (2·3%)	35 (2·6%)	132 (2·3%)	..
Marital status			..	..	..	<0·001
	Married or cohabiting		3169 (64·0%)	765 (55·5%)	3934 (62·2%)	
	Single		1096 (24·6%)	403 (24·7%)	1499 (24·7%)	..
	Divorced, separated, or widowed		1091 (11·4%)	534 (19·7%)	1625 (13·2%)	..
Economic activity			..	..	..	<0·001
	Employed		2838 (59·7%)	1022 (65·4%)	3860 (60·9%)	
	Unemployed		129 (3·0%)	74 (4·6%)	203 (3·3%)	..
	Other		2389 (37·4%)	606 (30·0%)	2995 (35·8%)	..
Tenure			..	..	..	<0·001
	Owner occupied		3770 (67·9%)	864 (52·0%)	4644 (64·5%)	
	Social renter		719 (13·4%)	418 (22·4%)	1137 (15·3%)	..
	Private or other		840 (18·7%)	398 (25·6%)	1238 (20·2%)	..
IMD quintiles			..	..	..	0·001
	Least deprived		1193 (20·9%)	284 (17·0%)	1477 (20·1%)	
	2nd		1167 (21·4%)	301 (17·1%)	1468 (20·5%)	..
	3rd		1109 (19·9%)	353 (20·5%)	1462 (20·1%)	..
	4th		990 (19·4%)	369 (22·0%)	1359 (20·0%)	..
	Most deprived		897 (18·4%)	395 (23·4%)	1292 (19·4%)	..
Adversities (ever)			..	..	..	<0·001
	0		405 (9·8%)	80 (5·1%)	485 (8·8%)	
	1 or 2		2132 (41·5%)	488 (31·3%)	2620 (39·3%)	..
	3 or 4		1925 (33·3%)	611 (34·3%)	2536 (33·6%)	..
	5 or more		885 (15·3%)	516 (29·3%)	1401 (18·3%)	..
Self-harm and suicidality (past year)						
	Suicidal thoughts		182 (3·6%)	170 (9·1%)	352 (4·8%)	<0·001
	Suicide attempt		18 (0·4%)	26 (1·4%)	44 (0·6%)	0·001
	Self-harm		49 (1·2%)	64 (4·0%)	113 (1·8%)	<0·001
Types of IPV experienced (ever)						
	Physical (any)		..	1154 (66·0%)	1154 (14·1%)	..
		Without injury	..	462 (29·1%)	462 (8·0%)	..
		With injury	..	692 (37·3%)	692 (6·2%)	..
	Sexual (any)		..	180 (9·5%)	180 (2·0%)	..
		Rape	..	134 (7·0%)	134 (1·5%)	..
	Emotional		..	1160 (66·4%)	1160 (14·2%)	..
	Economic		..	543 (28·4%)	543 (6·1%)	..
Number of types of IPV experiences (ever)						
	One type		..	788 (50·7%)	788 (10·8%)	..
	Two types		..	569 (32·0%)	569 (6·8%)	..
	Three types		..	269 (13·7%)	269 (2·9%)	..
All four types			..	76 (3·6%)	76 (0·8%)	..
Any IPV (past year)						
	IPV		..	292 (19·2%)	292 (4·1%)	..

Data are n (%). Percentages derived from the raw base sizes will not match percentages presented as analyses were weighted. IPV=intimate partner violence.

* Analytic sample of adults aged 16 years or older living in private households in England.

† p value for the overall association between each variable and experience of any IPV (ever).

Participants reporting IPV ever were less likely than the rest of the sample to be married or cohabiting; and more likely to be divorced, separated, or widowed, aged 35–54 years, and in employment. Some variation in the lifetime experience of IPV by ethnic group was evident (p=0·007). Rates appeared to be higher in people with a mixed, multiple, or other ethnicity (23·9%, 95% CI 17·3–32·2), and lower in the Asian or Asian British group (13·7%, 10·1–18·5); however, the sample sizes for these groups were small, and CIs around the estimates overlapped (data not shown in tables). Those with lifetime experience of IPV were more likely than those who had not experienced IPV to be living in rented accommodation and the most deprived quintile of neighbourhoods. They were twice as likely as the rest of the population (516 [29·3%] of 1702 *vs* 885 [15·3%] of 5356) to have faced five or more other adversities in their life, such as financial crises, redundancy, bereavement, and serious physical illness (table 1).

People with a lifetime history of IPV were 3 times more likely to have made a suicide attempt in the past year (26 [1·4%] of 1702) than those without any experience of IPV (18 [0·4%] of 5356; table 1). They were also approximately 3 times more likely in the past year to have self-harmed without suicidal intent (64 [4·0%] of 1702 *vs* 49 [1·2%] of 5356) and twice as likely to have had suicidal thoughts (170 [9·1%] of 1702 *vs* 182 [3·6%] of 5356). Among the population as a whole, emotional (1160 [14·2%] of 7058) and physical IPV ever (1154 [14·1%] of 7058) were the most commonly reported types, followed by economic (543 [6·1%]) and sexual (180 [2·0%]) IPV (table 1). Half of those who had any lifetime experience of IPV had experienced more than one type of IPV, and one in five had experienced at least one type in the past year (table 1).

Every indicator of IPV examined: any IPV ever; sexual, emotional, economic, and physical IPV ever; IPV with injury ever; multiple types of IPV ever; and past-year IPV, which was more prevalent in women than men (table 2 and appendix pp 3–4 for further gender disaggregation). Among women, 876 (19·6%) of 4177 had ever experienced emotional IPV, 878 (18·7%) had ever experienced physical IPV, and 425 (8·5%) ever experienced economic IPV, compared with 263 (8·6%) men for emotional IPV, 275 (9·3%) men physical IPV, and 95 (3·6%) men for economic IPV ever (table 2). The gender gap was widest for sexual IPV, where lifetime prevalence in women (3·7%) was about 10 times that of men (0·3%), and narrowest for past-year IPV, where prevalence in women (4·6%) and men (3·6%) was more similar (table 2). 15·9% of women and 5·0% of men had ever experienced more than one type of IPV (appendix p 3).

**Table 2:** Prevalence of each IPV indicator among people with and without experience of self-harm and suicidality in the past year, by gender*

		**No past-year suicidal thoughts, attempts, or self-harm**	**Past-year suicidal thoughts, attempts, or self-harm**	**Total (men)**	**No past-year suicidal thoughts, attempts, or self-harm**	**Past-year suicidal thoughts, attempts, or self-harm**	**Total (women)**	**No past-year suicidal thoughts, attempts, or self-harm**	**Past-year suicidal thoughts, attempts, or self-harm**	**Total**
**Suicidal thoughts in past year**										
Any IPV (ever)		411/2733 (14·6%)	48/141 (29·3%)	459/2874 (15·3%)	1120/3966 (26·0%)	122/211 (51·3%)	1242/4177 (27·2%)	1531/6699 (20·4%)	170/352 (40·6%)	1701/7051 (21·4%)
Types of IPV (ever)										
All physical IPV		247 (8·9%)	28 (17·8%)	275 (9·3%)	782 (17·7%)	96 (39·0%)	878 (18·7%)	1029 (13·4%)	124 (28·7%)	1153 (14·1%)
	Physical with physical injury	110 (3·8%)	14 (9·8%)	124 (4·1%)	492 (10·9%)	75 (28·1%)	567 (11·7%)	602 (7·4%)	89 (19·2%)	691 (8·0%)
All sexual IPV		6 (0·2%)	2 (1·8%)	8 (0·3%)	146 (3·4%)	26 (10·5%)	172 (3·7%)	152 (1·8%)	28 (6·3%)	180 (2·0%)
	Rape	3 (0·1%)	1 (1·5%)	4 (0·2%)	109 (2·4%)	21 (8·8%)	130 (2·7%)	112 (1·3%)	22 (5·3%)	134 (1·5%)
Emotional		226 (8·0%)	37 (21·8%)	263 (8·6%)	793 (18·4%)	104 (42·6%)	897 (19·6%)	1019 (13·3%)	141 (32·5%)	1160 (14·2%)
Economic		105 (3·4%)	13 (5·7%)	118 (3·6%)	380 (8·2%)	45 (15·5%)	425 (8·5%)	485 (5·8%)	58 (10·7%)	543 (6·1%)
Any IPV (past year)		81 (3·3%)	14 (10·3%)	95 (3·6%)	159 (3·9%)	38 (19·1%)	197 (4·6%)	240 (3·6%)	52 (14·8%)	292 (4·1%)
**Suicide attempt in past year**										
Any IPV (ever)		453/2860 (15·2%)	6/13 (39·6%)	459/2873 (15·3%)	1221/4144 (27·0%)	20/31 (58·4%)	1241/4175 (27·2%)	1674/7004 (21·2%)	26/44 (49·7%)	1700/7048 (21·4%)
Types of IPV (ever)										
All physical IPV		270 (9·2%)	5 (27·2%)	275 (9·3%)	860 (18·5%)	17 (43·2%)	877 (18·7%)	1130 (13·9%)	22 (35·8%)	1152 (14·1%)
	Physical with physical injury	120 (4·0%)	4 (27·2%)	124 (4·1%)	552 (11·5%)	14 (36·9%)	566 (11·7%)	672 (7·8%)	18 (32·6%)	690 (8·0%)
All sexual IPV		7 (0·2%)	1 (12·4%)	8 (0·3%)	168 (3·6%)	4 (19·0%)	172 (3·7%)	175 (2·0%)	5 (16·0%)	180 (2·0%)
	Rape	3 (0·1%)	1 (12·4%)	4 (0·2%)	127 (2·7%)	3 (15·8%)	130 (2·7%)	130 (1·4%)	4 (14·2%)	134 (1·5%)
Emotional		260 (8·6%)	3 (11·3%)	263 (8·6%)	878 (19·3%)	18 (53·4%)	896 (19·6%)	1138 (14·1%)	21 (33·9%)	1159 (14·2%)
Economic		117 (3·6%)	1 (1·9%)	118 (3·6%)	417 (8·5%)	8 (15·8%)	425 (8·5%)	534 (6·1%)	9 (9·3%)	543 (6·1%)
Any IPV (past year)		93 (3·6%)	2 (9·4%)	95 (3·6%)	190 (4·4%)	7 (34·8%)	197 (4·6%)	283 (4·0%)	9 (23·1%)	292 (4·1%)
**Self-harm in past year**										
Any IPV (ever)		443/2842 (14·9%)	16/32 (44·6%)	459/2874 (15·3%)	1195/4101 (26·7%)	48/81 (49·4%)	1243/4182 (27·2%)	1638/6943 (20·9%)	64/113 (47·7%)	1702/7056 (21·4%)
Types of IPV (ever)										
All physical IPV		267 (9·1%)	8 (23·0%)	275 (9·3%)	844 (18·4%)	35 (32·3%)	879 (18·7%)	1111 (13·8%)	43 (28·9%)	1154 (14·1%)
	Physical with physical injury	120 (4·0%)	4 (13·8%)	124 (4·1%)	542 (11·4%)	26 (24·5%)	568 (11·7%)	662 (7·8%)	30 (20·6%)	692 (8·0%)
All sexual IPV		6 (0·2%)	2 (7·1%)	8 (0·3%)	160 (3·5%)	12 (12·0%)	172 (3·7%)	166 (1·9%)	14 (10·2%)	180 (2·0%)
	Rape	2 (0·1%)	2 (7·1%)	4 (0·2%)	123 (2·6%)	7 (7·9%)	130 (2·7%)	125 (1·4%)	9 (7·6%)	134 (1·5%)
Emotional		252 (8·4%)	11 (25·6%)	263 (8·6%)	852 (19·0%)	45 (46·9%)	897 (19·6%)	1104 (13·7%)	56 (39·0%)	1160 (14·2%)
Economic		114 (3·5%)	4 (7·8%)	118 (3·6%)	405 (8·4%)	20 (13·7%)	425 (8·5%)	519 (6·0%)	24 (11·5%)	543 (6·1%)
Any IPV (past year)		91 (3·5%)	4 (9·4%)	95 (3·6%)	173 (4·1%)	24 (28·8%)	197 (4·6%)	264 (3·8%)	28 (21·6%)	292 (4·1%)

Data are n/N (%) or n (%). Percentages derived from the raw base sizes will not match percentages presented as analyses were weighted.

* See the appendix (pp 3–4) for extended analyses, including by number of types of IPV experienced, and base sizes. All IPV indicators were more common in those with suicidal thoughts, suicide attempt, non-suicidal self-harm, than in those without at p<0·05.

Among women who had attempted suicide in the past year, over half had ever experienced IPV (20 [58·4%] of 31), and a third of them (seven [34·8%]) had experienced IPV in the past year. In men who had attempted suicide in the past year, six (39·6%) of 13 had ever experienced IPV, and two (9·4%) had experienced this in the past year (table 2).

Although the prevalence of lifetime IPV was much higher in women than men, among both women and men the prevalence of self-harm and suicidality was higher in those who had experienced IPV than in those who had not. Interaction terms were tested and were all either small or there was no statistical evidence of a difference, indicating that the direction and strength of association between IPV and self-harm and suicidality were not statistically different in men and women in this dataset. For this reason, models were not gender stratified (table 2).

After adjustment for demographic factors (age, gender, ethnicity; version A models), the odds of a suicide attempt in the past year were 4·03 (95% CI 2·19–7·42) times higher in people with a lifetime history of IPV than in the rest of the population, and the adjusted odds ratios (aOR) for non-suicidal self-harm (3·06, 1·96–4·78) and suicidal thoughts (2·52, 1·94–3·27) were also raised (Table 3, Table 4, Table 5). Further adjustment for marital or cohabitation status, tenure, and area-level deprivation (version B models) attenuated the aORs somewhat. The final model (version C), which additionally adjusted for the other adversities in people's lives, reduced the aORs again, but all remained highly significant. In the final model, the aORs associated with lifetime experience of IPV were 2·82 (95% CI 1·54–5·17) for suicide attempt, 1·85 (1·39–2·46) for suicidal thoughts, and 2·20 (1·37–3·53) for self-harm. For those subjected to IPV in the year before interview, the aOR (version C) of past-year suicide attempt (3·79, 95% CI 1·90–7·53), suicidal thoughts (3·05, 2·04–4·56), and self-harm (3·04, 1·75–5·28) were particularly elevated (Table 3, Table 4, Table 5).

**Table 3:** Unadjusted and adjusted odds ratios for suicidal thoughts in the past year among people who had experienced each IPV indicator, compared with those who had not

			**Unadjusted OR**	aOR for demographics*	**aOR for demographics and socioeconomics**†	**aOR for demographics, socioeconomics, and adversities**‡
Any IPV (ever)			2·62 (2·04–3·37)	2·52 (1·94–3·27)	2·30 (1·76–3·01)	1·85 (1·39–2·46)
Type of IPV (ever)						
	All physical IPV		2·59 (1·95–3·44)	1·51 (1·05–2·19)	1·48 (1·01–2·18)	1·30 (0·89–1·89)
		Physical with injury§	2·96 (2·18–4·01)	..	..	..
	All sexual IPV		3·55 (2·09–6·03)	2·01 (1·07–3·78)	1·96 (1·02–3·74)	1·84 (0·96–3·54)
		Rape	4·16 (2·28–7·59)	..	..	..
	Emotional IPV		3·06 (2·35–3·99)	2·27 (1·61–3·21)	2·09 (1·47–2·96)	1·87 (1·32–2·64)
	Economic IPV		1·90 (1·34–2·69)	0·88 (0·56–1·38)	0·81 (0·51–1·28)	0·76 (0·48–1·21)
IPV count (ever)						
	One type		1·69 (1·17–2·44)	1·58 (1·08–2·30)	1·53 (1·05–2·24)	1·29 (0·88–1·91)
	Two types		3·89 (2·76–5·50)	3·89 (2·72–5·58)	3·42 (2·33–5·00)	2·72 (1·83–4·05)
	Three types		3·11 (2·01–4·81)	3·31 (2·12–5·19)	2·84 (1·77–4·53)	2·13 (1·32–3·42)
	All four types		3·64 (1·79–7·37)	3·89 (1·91–7·91)	3·08 (1·47–6·45)	2·08 (0·97–4·47)
	Any IPV (past year)		4·61 (3·19–6·66)	3·85 (2·61–5·68)	3·43 (2·30–5·11)	3·05 (2·04–4·56)

Data are OR (95% CI) or aOR (95% CI). aOR=adjusted odds ratio. IPV=intimate partner violence. OR=odds ratio.

* IPV indicators (either: any IPV, types of IPV, IPV count, or IPV in the past year) with adjustment for gender, age, and ethnicity; reference category: those not reporting the relevant IPV indicator.

† IPV indicators (either: any IPV, types of IPV, IPV count, or IPV in the past year) with adjustment for gender, age, ethnicity, marital status, tenure, and area-level deprivation.

‡ IPV indicators (either: any IPV, types of IPV, IPV count, or IPV in the past year) with adjustment for gender, age, ethnicity, marital status, tenure, area-level deprivation, and number of other adversities experienced.

§ Physical injuries included scratches, bruises, and broken bones.

**Table 4:** Unadjusted and adjusted odds ratios for suicide attempt in the past year among people who had experienced each IPV indicator, compared with those who had not

			**Unadjusted OR**	aOR for demographics*	**aOR for demographics and socioeconomics**†	**aOR for demographics, socioeconomics, and adversities**‡
Any IPV (ever)			3·98 (2·20–7·20)	4·03 (2·19–7·42)	3·58 (1·93–6·65)	2·82 (1·54–5·17)
Type of IPV (ever)						
	All physical IPV		3·02 (1·64–5.54)	1·52 (0·65–3·57)	1·44 (0·59–3·49)	1·25 (0·55–2·84)
		Physical with injury§	3·86 (2·11–7·07)	..	..	..
	All sexual IPV		7·83 (3·04–20·18)	4·57 (1·14–18·37)	3·97 (0·91–17·30)	3·65 (0·85–15·70)
		Rape	9·40 (3·28–26·96)	..	..	..
	Emotional IPV		4·12 (2·26–7·51)	2·98 (1·38–6·46)	2·75 (1·24–6·11)	2·37 (1·09–5·14)
	Economic IPV		2·36 (1·13–4·90)	0·91 (0·36–2·32)	0·73 (0·26–2·06)	0·68 (0·24–1·87)
IPV count (ever)						
	One type		3·02 (1·33–6·84)	2·72 (1·17–6·28)	2·71 (1·18–6·26)	2·31 (1·02–5·25)
	Two types		4·49 (2·14–9·40)	5·29 (2·53–11·07)	4·38 (2·04–9·39)	3·28 (1·57–6·85)
	Three types		5·73 (2·28–14·36)	8·23 (3·03–22·35)	6·64 (2·23–19·75)	4·71 (1·62–13·69)
	All four types		6·54 (2·10–20·32)	8·68 (2·48–30·38)	3·79 (1·05–13·68)	2·28 (0·62–8·33)
	Any IPV (past year)		7·88 (4·00–15·55)	5·59 (2·74–11·37)	4·45 (2·19–9·04)	3·79 (1·90–7·53)

Data are OR (95% CI) or aOR (95% CI). aOR=adjusted odds ratio. IPV=intimate partner violence. OR=odds ratio.

* IPV indicators (either: any IPV, types of IPV, IPV count, or IPV in past year) with adjustment for gender, age, and ethnicity; reference category: those not reporting the relevant IPV indicator.

† IPV indicators (either: any IPV, types of IPV, IPV count, or IPV in past year) with adjustment for gender, age, ethnicity, marital status, tenure, and area-level deprivation.

‡ IPV indicators (either: any IPV, types of IPV, IPV count, or IPV in past year) with adjustment for gender, age, ethnicity, marital status, tenure, area-level deprivation, plus number of other adversities experienced.

§ Physical injuries included scratches, bruises, and broken bones.

**Table 5:** Unadjusted and adjusted odds ratios for non-suicidal self-harm in the past year among people who had experienced each IPV indicator, compared with those who had not

			**Unadjusted OR**	aOR for demographics*	**aOR for demographics and socioeconomics**†	**aOR for demographics, socioeconomics, and adversities**‡
Any IPV (ever)			3·45 (2·27–5·23)	3·06 (1·96–4·78)	2·77 (1·76–4·37)	2·20 (1·37–3·53)
Type of IPV (ever)						
	All physical IPV		2·53 (1·62–3·95)	1·18 (0·60–2·34)	1·09 (0·54–2·24)	0·97 (0·49–1·92)
		Physical with injury§	3·09 (1·89–5·06)	..	..	..
	All sexual IPV		5·89 (2·79–12·43)	3·01 (1·14–7·92)	2·69 (1·02–7·12)	2·45 (0·90–6·65)
		Rape (ever)	5·87 (2·36–14·63)	..	..	..
	Emotional IPV (ever)		4·03 (2·61–6·21)	2·72 (1·44–5·13)	2·68 (1·38–5·22)	2·27 (1·17–4·43)
	Economic IPV (ever)		2·05 (1·24–3·37)	1·04 (0·54–2·00)	0·84 (0·40–1·76)	0·80 (0·41–1·58)
IPV count (ever)						
	One type		2·74 (1·54–4·87)	2·26 (1·23–4·17)	2·33 (1·26–4·31)	1·98 (1·05–3·74)
	Two types		4·04 (2·29–7·11)	3·67 (2·06–6·55)	3·05 (1·68–5·52)	2·32 (1·28–4·18)
	Three types		4·00 (2·05–7·81)	4·80 (2·33–9·90)	3·92 (1·81–8·47)	2·87 (1·36–6·09)
	All four types		6·34 (2·63–15·28)	6·34 (2·31–17·35)	3·57 (1·29–9·82)	2·33 (0·82–6·63)
	Any IPV (past year)		6·97 (4·08–11·91)	4·40 (2·52–7·67)	3·51 (2·01–6·16)	3·04 (1·75–5·28)

Data are OR (95% CI) or aOR (95% CI). aOR=adjusted odds ratio. IPV=intimate partner violence. OR=odds ratio.

* IPV indicators (either: any IPV, types of IPV, IPV count, or IPV in past year) with adjustment for gender, age, and ethnicity; reference category: those not reporting the relevant IPV indicator.

† IPV indicators (either: any IPV, types of IPV, IPV count, or IPV in past year) with adjustment for gender, age, ethnicity, marital status, tenure, and area-level deprivation.

‡ IPV indicators (either: any IPV, types of IPV, IPV count, or IPV in past year) with adjustment for gender, age, ethnicity, marital status, tenure, area-level deprivation, plus number of other adversities experienced.

§ Physical injuries included scratches, bruises, and broken bones.

Sexual and emotional IPV ever appeared to be stronger predictors of suicidality and self-harm than physical and economic IPV ever. The unadjusted OR of a suicide attempt in the past year was 9·40 (95% CI 3·28–26·96) times higher in people who had ever been raped by a partner compared with those who had not, and 7·83 (3·04–20·18) times higher in victims of any sexual IPV ever compared with people who had not experienced sexual IPV. Likewise, the unadjusted OR of a suicide attempt appeared higher for those who had ever experienced physical IPV with injury (3·86, 2·11–7·07), than for those who had experienced any physical IPV ever (3·02, 1·64–5.54). Another potential indication of a dose–response relationship between exposure to IPV ever and past-year suicidality and self-harm outcomes comes from examining the number of types of IPV experienced. Those who had ever experienced multiple IPV types tended to have higher odds of past year suicidal thoughts, suicide attempt, and self-harm than those with lifetime experience of one IPV type; however, some of these estimates were imprecise and some 95% CIs overlapped.

## Discussion

Although associations between IPV and mental disorder are well established, this analysis provides urgently needed evidence on the association between IPV and suicidality and self-harm. Our results confirm that those who have experienced IPV are far more likely to be women than men, and they show for the first time, to our knowledge, that a strong association is evident in both men and women and adults of all ages. Associations are particularly pronounced for those who recently experienced IPV, those who were ever subjected to sexual and emotional IPV, those who ever experienced physical injury, and those who have experienced multiple forms of IPV, indicating a dose–response relationship. Our findings are consistent with the high rates of suicidality disclosed by those using domestic violence support services.^8^

This study is, to our knowledge, the first to examine different IPV types (including emotional IPV) and self-harm and suicidality in a national probability sample, allowing comparisons by gender and across the adult age range. These analyses are among the first to consider the relationship between IPV and suicidality, adjusting for multiple adversities. However, limitations must be acknowledged. Although lifetime IPV was examined as a predictor of recent suicidality, APMS is cross-sectional and unable to establish causality. People with experience of suicidality could have increased risk of subsequent violence victimisation;^22^ longitudinal studies are better able to disentangle this.^10^ Although adjustments were made for multiple adversities, the elevated prevalence of suicidality in those exposed to IPV might have been, at least in part, explained by factors that studies such as this could not adjust for.^20^ The number of participants reporting IPV was too small for robust analysis by ethnic group and intersectional inequalities. Few men in the sample reported sexual IPV, severely limiting the scope for gender disaggregation. Further consideration of gender identities was not possible as participants were coded on the survey as women or men. Although some consideration was made of clustering in types of IPV and severity of physical (with or without injury) and sexual (rape or any sexual contact) IPV, we lacked comprehensive information on repetition, and our measure of economic IPV might have not captured this multidimensional form of abuse.^23^ As a household sample, people living in refuges, prisons, or other institutional settings, or who were experiencing homelessness were out of its scope, and these groups are likely to be at higher risk of both IPV and self-harm and suicidality.^24^ Although some information was self-completed, under-reporting of stigmatised experiences remains possible, especially where participants were living with a violent partner at the time of the interview.^15^ Missing data were unlikely to be missing at random, possibly introducing bias, although the extent was minimal.

The survey was done in 2014; analyses of police and domestic service provider data indicate that the COVID-19 context could have influenced the prevalence of IPV.^25^ The nature of the association between suicidality and IPV might have changed, given restrictions due to lockdowns and changes in access to social support and services.^26^ During the COVID-19 pandemic, survey data collection on domestic violence and self-harming behaviours effectively ceased.^27^ Reliance on data from police, specialised domestic abuse service providers, and health services provides a partial picture. It is key that survey data collection on both IPV and self-harm resumes, that this includes longitudinal designs better able to distinguish confounders and mediators, and that data collected are swiftly made accessible to researchers.^28^

This study shows that IPV might be more common in England than previously thought. The lifetime prevalence found by this study (21·4%) was higher than that from the 2019 CSEW (17·2%), the official source for IPV prevalence used by the UK Government.^29^ The CSEW estimate is lower despite it including more IPV types (stalking, indecent exposure, and threat) and a younger sample (ages 16–74 years). APMS spanned the full adult age range and found lifetime reports of IPV were lower in those aged 75 years and older, probably reflecting issues of recall and healthy survivor bias. The higher IPV reporting in APMS than in CSEW might also stem from survey framing, with some participants perhaps more reluctant to report certain experiences in the context of a crime survey rather than a health survey. Official IPV prevalence figures might be underestimates.

One in three women who had attempted suicide in the past year was a recent IPV victim (compared with one in 20 women in the rest of the population). This was also the case for one in ten men who made a suicide attempt. This finding supports routine enquiry about IPV in health care and other settings when someone presents having self-harmed or in suicidal distress, with steps taken to protect those who are exposed.

Suicide attempts and self-harming behaviours are key risk indicators for subsequent suicide and are relevant to suicide prevention.^30^ Suicide is rarely the consequence of a single risk factor or event, but could result from a cumulation and interaction of multiple factors.^26^ These analyses show that those subject to IPV were more likely to live in precarity: in rented accommodation and the most deprived neighbourhoods. Socioeconomic insecurity and lack of resources have been identified both as risks for suicidality and as prolonging the duration of domestic violence.^31^ IPV victims were twice as likely as those who had not experienced IPV to have faced five or more major adversities in their lives in addition to IPV, including financial crises, bereavement, job loss, and illness. Poverty and IPV share aspects of entrapment, identified as a mechanism in the transition from suicidal thoughts and non-suicidal self-harm to suicide attempt and suicide.^32^ Suicide reduction interventions in the context of IPV need to address people's wider social and economic context. However, even when controlling for wider adversities, being exposed to IPV still confers additional and independent risk for self-harm and attempted suicide. The interactions between IPV, other adversities, and suicidality are likely to be complex. For some who self-harm, IPV might be a risk acting alongside many other risks, whereas for others it might be a more direct cause. Interventions designed to reduce the prevalence and duration of IPV, could also have the potential to reduce suicide in the population and should feature both in national suicide prevention strategies and individual-level suicide risk assessments and risk reduction plans.^33^

For more on **Who national suicide prevention strategies** see https://apps.who.int/iris/handle/10665/279765

For the **Data Access Request Service at NHS Digital** see https://digital.nhs.uk/services/data-access-request-service-dars

## Data sharing

The deidentified APMS dataset, data dictionary, protocol, participant materials, and full documentation are lodged with the UK Data Service archive. Permission to use the dataset for this analysis was obtained from NHS Digital. Requests for further use should be made to the Data Access Request Service at NHS Digital .

**This online publication has been corrected. The corrected version first appeared at thelancet.com on Aug 1, 2022**

## Declaration of interest

LA chairs England's cross-government National Suicide Prevention Strategy Advisory Group. DK is funded by Wellcome Trust and the Elizabeth Blackwell Institute Bristol. All other authors declare no other competing interests.
