# Supplementary material for: Intimate partner violence, suicidality, and self-harm: a probability sample survey of the general population in England
Source: Lancet Psychiatry. 2022 Jul;9(7):574–83. doi: 10.1016/S2215-0366(22)00151-1 (PMC9630147; doi:10.1016/S2215-0366(22)00151-1)
Supplement: Supplementary appendix [file mmc1.pdf]

THE LANCET
Psychiatry

Supplementary appendix

This appendix formed part of the original submission and has been peer reviewed. We post it as supplied by the authors.

Supplement to: Sally McManus, Sylvia Walby, Estela Capelas Barbosa.
Estela Capelas Barbosa. Intimate partner violence, suicidality, and self-harm: a probability sample survey of the general population in England.
Lancet Psychiatry 2022; published online June 7. https://doi.org/10.1016/S2215-0366(22)00151-1.

Intimate partner violence, suicidality, and self-harm: a probability sample survey of the general population in England

Appendix

Table of contents

1. Search terms used to identify relevant existing research page 2
2. Weighting strategy used on the Adult Psychiatric Morbidity Survey page 2
3. Gender-stratified prevalence estimates page 3
4. Gender-violence interactions page 5

1. Search terms used to identify relevant existing research

Any of: ‘intimate partner violence’, ‘intimate partner abuse’, ‘domestic violence’, ‘domestic abuse’,
AND any of: ‘suicidality’, ‘suicide’, ‘suicidal’, ‘self-harm’, ‘self-injury’.

2. Weighting strategy used on the Adult Psychiatric Morbidity Survey (APMS)

The percentages and odds ratios presented in the paper are weighted to account for likelihood of selection and non-response. Bases are presented unweighted to show the true number of responding participants. Weights were applied during data analysis to render results generalisable to the general population. The weights address potential biases that could result from differential selection probabilities (resulting, for example, from one person being selected from each household, leading to the systematic under representation of people living in multi-person households in the raw sample). The weights also correct for different patterns of non-response (for example, correcting for lower response from, for example, more deprived neighbourhoods, particular regions of the country, younger people, and men). The weights were created by the survey term based at the National Centre for Social Research and form part of the archived survey dataset. The approach to developing the survey weights is detailed fully in the APMS report[1], and reproduced here for reference.

Weighting occurred in four steps. First, address selection weights (wt1) were applied to take account of the differential selection probabilities of addresses. For each of the 698 sampled PSUs, the weight was calculated as follows: wt1 = total addresses on PAF / (698 x number of sampled addresses per PSU). All addresses in the same PSU were assigned the same weight.

Second, to reduce household non-response bias, a household level weight was calculated from a logistic regression model using interviewer observation and area-level variables (collected from Census 2011 data) available for responding and non-responding households. The dependent variable was whether the household responded or not. The independent variables considered for inclusion in the model were the presence of any physical barriers for entry to the property (e.g. a locked common entrance or the presence of security staff), Government Office Region (GOR), Index of Multiple Deprivation 2010 (IMD 2010) quintiles, population density (number of persons per hectare), percentage of households owner-occupied, and the percentage of adults in a non-manual occupation. Not all the variables were retained for the final model: variables not significantly related to the propensity of households to respond were dropped from the analysis. The variables significantly associated with response were: GOR, whether there were entry barriers to the selected address, the percentage of households owner-occupied and population density. The model shows that the propensity for a household to respond was lower in Yorkshire and Humberside, East of England, and in inner and outer London (relative to the North East), higher for households with no physical barriers for entry to the property, higher in areas where a relatively high percentage of households were owner-occupied and lower in areas with a relatively high population density. The non-response weight (wt2) for each eligible household was calculated as the inverse of the probability of response estimated from the final model.

[1] McManus S, Bebbington P, Jenkins R, Brugha T. (eds.) (2016) *Mental health and wellbeing in England: Adult Psychiatric Morbidity Survey 2014*. Leeds: NHS Digital. https://files.digital.nhs.uk/pdf/q/3/mental_health_and_wellbeing_in_england_full_report.pdf

Third, selection weights (wt3) were applied to take account of the different probabilities of selecting participants in different sized households. The weight was equal to the number of adults (16+) in the household, the inverse of the probability of selection. The composite weight for selection and participation was calculated as the product of the weights from the previous stages: wt4 = wt1 x wt2 x wt3.

The final stage of the weighting was to adjust the composite weight (wt4) using calibration weighting. Calibration takes an initial weight (in this case wt4) and adjusts it to given control totals. The process generates a weight which produces survey estimates that exactly match the population for the specific characteristics used in the adjustment. Calibration reduces any residual non-response bias and any impact of sampling and coverage error for the measures used in the adjustment. The population control totals used were the ONS 2014 mid-year population estimates for age-by-sex and region. After calibration, the APMS 2014 weighted data matches the estimated population in terms of age-by-sex and region.

3. Gender-stratified prevalence estimates

The prevalence of intimate partner violence (IPV) varied between men and women. Tables A1, A2 and A3 summarise these differences for each IPV indicator examined in the paper. The prevalence is presented for all adults in the sample, for all men and all women, and for men, women, and all adults reporting each of the outcomes: past-year experience of suicidal thoughts, suicide attempt, and non-suicidal self-harm.

Table A1. Prevalence of each IPV indicator among people with and without suicidal thoughts in the past year, by gender

	**Men**			**Women**			**Total [a]**		
	**No suicidal thoughts** (n=2733)	**Suicidal thoughts** (n=141)	**All men** (n=2874)	**No suicidal thoughts** (n=3966)	**Suicidal thoughts** (n=211)	**All women** (n=4177)	**No suicidal thoughts** (n=6699)	**Suicidal thoughts** (n=352)	**All adults** (n=7051)
	%	%	**%**	%	%	**%**	%	%	**%**
**Any IPV (ever)**	**14·6**	**29·3**	**15·3**	**26·0**	**51·3**	**27·2**	**20·4**	**40·6**	**21·4**
**Types of IPV (ever)**									
Physical - all	8·9	17·8	9·3	17·7	39·0	18·7	13·4	28·7	14·1
Physical with physical injury	3·8	9·8	4·1	10·9	28·1	11·7	7·4	19·2	8·0
Sexual - all	0·2	1·8	0·3	3·4	10·5	3·7	1·8	6·3	2·0
Rape	0·1	1·5	0·2	2·4	8·8	2·7	1·3	5·3	1·5
Emotional	8·0	21·8	8·6	18·4	42·6	19·6	13·3	32·5	14·2
Economic	3·4	5·7	3·6	8·2	15·5	8·5	5·8	10·7	6·1
**Number of IPV types (ever)**									
None	85·4	70·7	84·7	74·0	48·7	72·8	79·6	59·4	78·6
One type	10·2	14·1	10·4	11·2	12·9	11·3	10·7	13·5	10·8
Two types	3·1	12·9	3·6	9·3	24·0	10·0	6·3	18·6	6·8
Three types	1·2	2·0	1·3	4·2	10·9	4·5	2·8	6·5	2·9
All four types	0·1	0·3	0·1	1·3	3·5	1·4	0·7	2·0	0·8
**Any IPV (past year)**	**3·3**	**10·3**	**3·6**	**3·9**	**19·1**	**4·6**	**3·6**	**14·8**	**4·1**

[a] All IPV indicators were more common in those with past year suicidal thoughts, suicide attempt, non-suicidal self-harm, than in those without at $p<0·05$.

Table A2. Prevalence of each IPV indicator among people who had and had not made a suicide attempt in the past year, by gender

	Men			Women			Total [a]		
	**No suicide attempt** (n=2860)	**Suicide attempt** (n=13)	**All men** (n=2873)	**No suicide attempt** (n=4144)	**Suicide attempt** (n=31)	**All women** (n=4175)	**No suicide attempt** (n=7004)	**Suicide attempt** (n=44)	**All adults** (n=7048)
	%	%	**%**	%	%	**%**	%	%	**%**
**Any IPV (ever)**	**15·2**	**39·6**	**15·3**	**27·0**	**58·4**	**27·2**	**21·2**	**49·7**	**21·4**
**Types of IPV (ever)**									
Physical - all	9·2	27·2	9·3	18·5	43·2	18·7	13·9	35·8	14·1
Physical with physical injury	4·0	27·2	4·1	11·5	36·9	11·7	7·8	32·6	8·0
Sexual - all	0·2	12·4	0·3	3·6	19·0	3·7	2·0	16·0	2·0
Rape	0·1	12·4	0·2	2·7	15·8	2·7	1·4	14·2	1·5
Emotional	8·6	11·3	8·6	19·3	53·4	19·6	14·1	33·9	14·2
Economic	3·6	1·9	3·6	8·5	15·8	8·5	6·1	9·3	6·1
**Number of IPV types (ever)**									
0	84·8	60·4	84·7	73·0	41·6	72·8	78·8	50·3	78·6
1	10·3	28·3	10·4	11·3	5·2	11·3	10·8	15·9	10·8
2	3·6	9·4	3·6	9·7	37·4	10·0	6·7	24·4	6·8
3	1·3	1·9	1·3	4·5	11·8	4·5	2·9	7·2	2·9
All 4	0·1	-	0·1	1·4	4·0	1·4	0·8	2·2	0·8
**Any IPV (past year)**	**3·6**	**9·4**	**3·6**	**4·4**	**34·8**	**4·6**	**4·0**	**23·1**	**4·1**

[a] All IPV indicators were more common in those with past year suicidal thoughts, suicide attempt, non-suicidal self-harm, than in those without at $p<0·05$.

Table A3. Prevalence of each IPV indicator among people who had and had not self-harmed in the past year, by gender

	Men			Women			Total [a]		
	**Not self-harmed** (n=2842)	**Self-harmed** (n=32)	**All men** (n=2874)	**Not self-harmed** (n=4101)	**Self-harmed** (n=81)	**All women** (n=4182)	**Not self-harmed** (n=6943)	**Self-harmed** (n=113)	**All adults** (n=7056)
	%	%	**%**	%	%	**%**	%	%	**%**
**Any IPV (ever)**	**14·9**	**44·6**	**15·3**	**26·7**	**49·4**	**27·2**	**20·9**	**47·7**	**21·4**
**Types of IPV (ever)**									
Physical - all	9·1	23·0	9·3	18·4	32·3	18·7	13·8	28·9	14·1
Physical with physical injury	4·0	13·8	4·1	11·4	24·5	11·7	7·8	20·6	8·0
Sexual - all	0·2	7·1	0·3	3·5	12·0	3·7	1·9	10·2	2·0
Rape	0·1	7·1	0·2	2·6	7·9	2·7	1·4	7·6	1·5
Emotional	8·4	25·6	8·6	19·0	46·9	19·6	13·7	39·0	14·2
Economic	3·5	7·8	3·6	8·4	13·7	8·5	6·0	11·5	6·1
**Number of IPV types (ever)**									
0	85·1	55·4	84·7	73·3	50·6	72·8	79·1	52·3	78·6
1	10·0	33·9	10·4	11·3	10·9	11·3	10·7	19·4	10·8
2	3·6	4·2	3·6	9·6	25·6	10·0	6·6	17·7	6·8
3	1·2	4·8	1·3	4·4	9·2	4·5	2·9	7·5	2·9
All 4	0·1	1·7	0·1	1·4	3·9	1·4	0·7	3·1	0·8
**Any IPV (past year)**	**3·5**	**9·4**	**3·6**	**4·1**	**28·8**	**4·6**	**3·8**	**21·6**	**4·1**

[a] All IPV indicators were more common in those with past year suicidal thoughts, suicide attempt, non-suicidal self-harm, than in those without at $p<0·05$.

4. Gender-violence interaction terms

Into models with a suicidality or self-harm outcome and gender and violence indicators as predictors, an interaction term was also entered for violence and gender (for example, 'PhysicalViolence*RespondentGender' as a predictor of suicidal thoughts; or 'SexualViolence*RespondentGender' as a predictor of self-harm).

In general, the patterns of association between experience of violence and suicidality and self-harm outcomes did not differ between men and women in a statistically significant way. That is, IPV predicted suicidal thoughts, suicide attempts and self-harm in both men and women and to a broadly similar degree. There was an indication (p=0·002) that physical violence with injury had a stronger association with suicidal thoughts in women than in men, although the effect size for this difference was small (aOR 1·2) and not significant for suicide attempt or self-harm. There were also indications that sexual IPV ever was a stronger predictor of suicide attempt and self-harm in men than women, however it should be noted that sexual IPV ever was reported by just eight men in the sample (compared with 172 women), and thus the sample was insufficient to examine this. The results presented below relate to testing these interactions in simple models, adding interaction terms to the fully adjusted models also did not indicate a statistical difference between men and women in the association between IPV and suicidality.

Table B1. Odds ratios and significance of violence-sex interaction terms as predictors of suicidality and self-harm in the past year, adjusting for sex and violence

	**Suicidal thoughts in past year**		**Suicide attempt in past year**		**Self-harm in past year**	
	AOR	p value	AOR	p value	AOR	p value
**Any IPV (ever)*Sex**	1·2	0·483	0·7	0·577	0·6	0·245
**Types of IPV(ever)*Sex**						
Physical*Sex	1·3	0·359	0·9	0·458	0·7	0·497
Physical with physical injury*Sex	1·2	0·002	1·1	0·534	1·1	0·295
Sexual*Sex[a]	0·4	0·347	0·1	0·005	0·1	0·025
Rape*Sex[a]	0·3	0·309	0·0	0·004	0·0	0·007
Emotional*Sex	1·0	0·976	1·2	0·793	1·0	0·992
Economic*Sex	1·4	0·666	0·5	0·344	0·7	0·638
**Any IPV (past year)*Sex**	1·7	0·331	2·6	0·196	3·4	0·067

[a] Note that 8 men and 172 women reported experience of sexual IPV, so these results should be interpreted with caution.
